# Development of a Supportive Parenting App to Improve Parent and Infant Outcomes in the Perinatal Period: Development Study

**DOI:** 10.2196/27033

**Published:** 2021-12-24

**Authors:** Shefaly Shorey, Thiam Chye Tan, Thilagamangai, Jancy Mathews, Chun Yan Yu, Siew Hoon Lim, Luming Shi, Esperanza Debby Ng, Yiong Huak Chan, Evelyn Law, Cornelia Chee, Yap Seng Chong

**Affiliations:** 1 Alice Lee Centre for Nursing Studies Yong Loo Lin School of Medicine National University of Singapore Singapore Singapore; 2 Mount Elizabeth Novena Specialist Centre Singapore Singapore; 3 Division of Nursing KK Women’s and Children’s Hospital Singapore Singapore; 4 National University Polyclinics Singapore Singapore; 5 Raffles Medical Group Singapore Singapore; 6 Singapore General Hospital Singapore Singapore; 7 Singapore Clinical Research Institute Singapore Singapore; 8 Biostatistics Unit Yong Loo Lin School of Medicine National University of Singapore Singapore Singapore; 9 National University Hospital Singapore Singapore; 10 Yong Loo Lin School of Medicine National University of Singapore Singapore Singapore

**Keywords:** depression, development, education, parent, perinatal, support, telehealth, mobile phone

## Abstract

**Background:**

The transition to parenthood can be challenging, and parents are vulnerable to psychological disorders during the perinatal period. This may have adverse long-term consequences on a child’s development. Given the rise in technology and parents’ preferences for mobile health apps, a supportive mobile health intervention is optimal. However, there is a lack of a theoretical framework and technology-based perinatal educational intervention for couples with healthy infants.

**Objective:**

The aim of this study is to describe the Supportive Parenting App (SPA) development procedure and highlight the challenges and lessons learned.

**Methods:**

The SPA development procedure was guided by the information systems research framework, which emphasizes a nonlinear, iterative, and user-centered process involving 3 research cycles—the relevance cycle, design cycle, and rigor cycle. Treatment fidelity was ensured, and team cohesiveness was maintained using strategies from the Tuckman model of team development.

**Results:**

In the relevance cycle, end-user requirements were identified through focus groups and interviews. In the rigor cycle, the user engagement pyramid and well-established theories (social cognitive theory proposed by Bandura and attachment theory proposed by Bowlby) were used to inform and justify the features of the artifact. In the design cycle, the admin portal was developed using Microsoft Visual Studio 2017, whereas the SPA, which ran on both iOS and Android, was developed using hybrid development tools. The SPA featured knowledge-based content, informational videos and audio clips, a discussion forum, chat groups, and a frequently asked questions and expert advice section. The intervention underwent iterative testing by a small group of new parents and research team members. Qualitative feedback was obtained for further app enhancements before official implementation. Testing revealed user and technological issues, such as web browser and app incompatibility, a lack of notifications for both administrators and users, and limited search engine capability.

**Conclusions:**

The information systems research framework documented the technical details of the SPA but did not take into consideration the interpersonal and real-life challenges. Ineffective communication between the health care research team and the app developers, limited resources, and the COVID-19 pandemic were the main challenges faced during content development. Quick adaptability, team cohesion, and hindsight budgeting are crucial for intervention development. Although the effectiveness of the SPA in improving parental and infant outcomes is currently unknown, this detailed intervention development study highlights the key aspects that need to be considered for future app development.

## Introduction

### Background

In recent years, depression has displaced many chronic diseases to be the leading cause of disability worldwide and the main contributor to the overall global burden of diseases [1]. Although depression can occur at any point in life, women and men are at increased risk of depression in the postpartum period [2,3]. The transition to parenthood, coupled with emotional, physiological, hormonal, and psychosocial changes experienced during childbirth, could have increased women’s susceptibility to postpartum depression [4-6]. The psychological state of women could influence their partners’ psychological well-being because maternal postpartum depression is a known predictor of paternal postpartum depression, affecting 24%-50% of all fathers [7]. Fathers also have the highest risk of developing paternal postpartum depression 3 to 6 months after childbirth [8,9]. Moreover, research has shown the ripple effects of postpartum depression adversely affecting an infant’s subsequent cognitive, physical, social-emotional, and behavioral development over the years [10,11]. Children of mothers who were depressed were found to score lower in intellectual and motor development, and they tend to display insecure attachment, behavioral problems, poor emotional regulation and academic performances, and are at an increased risk of developing psychiatric disorders [12-14]. In addition, a child’s internalizing and externalizing behavior problems are significantly related to paternal postpartum depression [15].

Given the potential widespread consequences of postpartum depression on the family unit, there is a strong impetus for couple-based interventions to curb postpartum depression. However, to date, the cause of postpartum depression among parents remains inconclusive, thus inhibiting the development of direct intervention. Instead, research has suggested a multifactorial etiology, as there has been evidence that various psychosocial variables, such as life stress, marital conflict, childcare stress, parenting self-efficacy, poor childcare knowledge, the lack of social support, and antenatal anxiety, are key predictors of postpartum depression [5,6]. Hence, a perinatal intervention targeting these predictors may help to alleviate feelings of stress and depression among parents. Reviews on previous interventions implemented during the perinatal period have indicated the need for more multimodal family-oriented educational programs that focus on parental physical, emotional, and social well-being [16-18]. Furthermore, theory-based educational interventions are scarce, and current interventions are often compartmentalized as antenatal or postpartum [16-19] and focus on specific groups of parents such as those with infants in the neonatal intensive care unit or with behavioral problems [16-18,20,21]. Therefore, there is an urgent need for a comprehensive perinatal educational intervention for parents with healthy infants to enhance parental psychological well-being and infant developmental outcomes.

### Value of Technology

Technology-enhanced health care and telemedicine have been gaining popularity because of their better accessibility, flexibility, cost-effectiveness, and ability to provide patient privacy and reduce stigmatization [22,23]. The rapid proliferation in technology-based health care is enabled by the increased use and advancements of mobile apps, wearable devices, live audiovisual communication, and telecommunication. The value of telehealth care and technology-based interventions has been further amplified during the ongoing COVID-19 pandemic as most households worldwide have faced lockdown restrictions, quarantine measures, and social isolation, which limit couples’ access to help and support [24]. Moreover, such technology-based interventions are especially crucial in a conservative society such as Singapore’s, where traditional views and postpartum practices (eg, home confinement) as well as the stigmatization of mental health disorders restrict parents from seeking help in the postpartum period [25,26].

Previous studies examining the needs of fathers and mothers during the perinatal period have also highlighted parents’ preferences for technology-based educational programs, especially mobile health (mHealth) apps [26,27]. mHealth apps have a relatively new edge in health care innovation that allows the delivery of health care and dissemination of educational information [27]. In 2010, more than 200 million mHealth apps were downloaded, and approximately 70% of the population worldwide has access to at least one mHealth app [28]. mHealth pregnancy-related apps are particularly popular among young parents, suggesting a shift in patient empowerment in terms of maternity care [29,30]. However, parents have expressed difficulty in finding pregnancy apps that provide localized and individualized advice and feedback from health care professionals [27,30]. Current literature has also revealed that most of the available mHealth apps are not validated or developed by health care professionals [31], thus calling into question the trustworthiness and credibility of the information provided.

Given the benefits of mHealth apps and the paucity and limitations of current pregnancy-related mHealth apps, this study aims to develop the Supportive Parenting App (SPA), a theory-based perinatal educational mobile app for parents, and to subsequently examine its effectiveness in preventing postpartum depression and enhancing parenting self-efficacy, perceived social support, parent-child bonding, and physical and social-emotional development of an infant. According to the Singapore Infocomm Media Development Authority [32], 98% of households in Singapore in 2019 had internet access and 89% of individuals were internet users. In addition, the smartphone penetration rate of 82% in Singapore is one of the highest in the Asia Pacific region [33]. Therefore, technology is available in most households to support the implementation of the SPA during the perinatal period.

Detailed intervention development studies are limited because of research funding priorities that focused on efficacy or effectiveness studies [34]. However, a comprehensive, systematic, and transparent reporting of intervention development is necessary to help readers understand the challenges and benefits of different intervention development approaches and allow retrospective assessments to be performed to understand how different development approaches may affect the effectiveness of interventions. Therefore, this study aims to provide an insight into the SPA development process and highlight the challenges and lessons faced during the SPA development to improve future mHealth studies.

### Project Overview

The SPA project was a multisite study that was conducted in tertiary public hospitals in Singapore. The intervention was delivered to both parents (fathers and mothers), and they were granted access to the SPA during the perinatal period (from the viability age of 24 weeks of gestation to 6 months after childbirth) when the chances of developing antenatal or postpartum depression were very high. The parents were able to access the mobile app anytime and anywhere. The SPA featured educational materials, a discussion forum, chat groups with peer volunteers, and advice from health care professionals. The inclusion criteria were as follows: (1) parents with a female partner who scored ≥9 on the Edinburgh Postnatal Depression Scale, (2) those aged ≥21 years, (3) those who were able to read and speak English, (4) those who had a low-risk pregnancy with more than 24 weeks of gestation (age of viability), and (5) those who had a smartphone with internet access. The exclusion criteria were as follows: parents who (1) had physical or mental disorders that would interfere with their ability to participate in the study; (2) had a high-risk pregnancy, including placenta previa major, preeclampsia, and pregnancy-induced hypertension; or (3) gave birth to a stillborn baby or a newborn with congenital anomalies or medical complications, including pathological jaundice that required special care in hospital. Mothers aged ≥21 years who had previously recovered from postpartum depression were recruited and trained by a psychiatrist to become peer volunteers to befriend and provide adequate web-based support to the parents.

The aims of this research project are to (1) develop a theory-based SPA intervention for parents across the perinatal period; (2) examine the intervention’s effectiveness in reducing parental postpartum depression and anxiety and encouraging parenting self-efficacy, help-seeking behavior, parent-infant bonding, parenting satisfaction, and infant developmental outcomes during the perinatal period up to 1 year after childbirth; (3) evaluate the SPA’s cost-effectiveness compared with standard perinatal care across major restructured hospitals; and (4) examine SPA user experiences. However, this paper only focuses on the SPA intervention development process, the challenges faced by the research team, and the lessons learned.

## Methods

### Information Systems Research Framework

**Figure 1:**
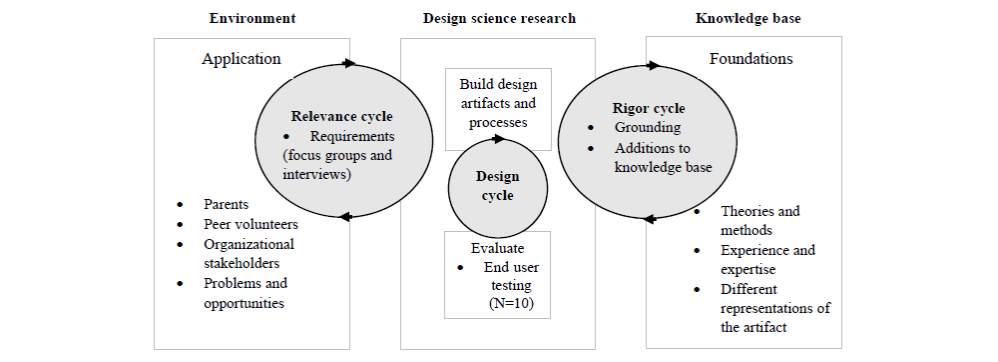
Adaptation of the information systems research framework.

The information systems research (ISR) framework, which promotes an iterative, user-centered, nonlinear design process was used to guide the development of the SPA [35]. The ISR framework is commonly used in the creation of technological artifacts and comprises 3 research cycles (relevance cycle, design cycle, and rigor cycle) that link 3 research domains (knowledge base, design science research, and environment). The relevance cycle involves understanding the end users’ needs and requirements through feedback and focus groups. The design cycle is where the artifact is developed and evaluated, and the rigor cycle is where theories, past methods, and representations of the artifacts contribute to the current design and development of the new artifact [35]. This study’s adaptation of the ISR framework [35] is presented in Figure 1.

### Treatment Fidelity

To establish treatment fidelity, the strategies proposed by Bellg et al [36] and Eaton et al [37] for intervention design, training of providers, intervention delivery, and receipt of intervention were adopted. During the intervention design phase, intervention fidelity was ensured by using a theoretical framework (social cognitive theory) as the foundation of the intervention and by preparing a protocol for the intervention. In anticipation of provider and peer volunteer dropout, backup providers and volunteers with the requisite skills were on constant standby. To ensure consistency, delivery of intervention materials (eg, knowledge-based content and expert advice) was standardized by providing briefings and issuing providers and peer volunteers a handbook on the intervention procedures. In terms of receipt of intervention, participants’ frequency of access to the app and the use of audio and video materials and peer discussion forum were monitored and recorded through a back-end admin portal. Weekly reminders were sent to parents to reinforce the use of the intervention by accessing the SPA.

### Team Development

As the SPA development process involved stakeholders across various departments, immense collaborative effort and a high-performing team were crucial to ensure consistent progress and adherence to the project timeline. The Tuckman model of team development [38] highlighted different phases of team development—forming (initial meeting and beginning of collective work), storming (dealing with confusion and conflict over decision-making and roles), norming (accepting goals, roles, and work positively), performing (focusing on achieving goals and obtaining personal growth), and adjourning (recognizing and celebrating achievements). Although all phases are necessary for successful team building, problem solving, work planning, and delivering of results, the team leader plays a critical role in regulating the team dynamics and ensuring effective workflow by applying appropriate conflict resolution strategies as recommended by Tuckman [38].

## Results

### Relevance Cycle

The end-user environment and requirements were obtained through a focus group session with new parents. In all, 3 pairs of parents representing each major ethnic group in Singapore (Chinese, Malay, and Indian) were interviewed on their informational and perinatal educational needs and their expectations of the SPA. These parents were not included in the subsequent main study. In addition, because the objectives and components of the SPA were similar to those of the research team’s previous research projects—*Home-but-not-Alone* (an mHealth app-based postnatal educational program) and the *Peer-Support Intervention Program*—user requirements were obtained from the projects’ focus groups and interviews [39,40]. Previous local research among Singaporean mothers who only received standard routine hospital care indicated a need for more support after childbirth, such as having peer volunteers to talk to, more follow-up appointments with clinicians, and having an educational parenting mobile app catered to parents in Singapore [40]. Mothers who received the intervention requested the inclusion of individualized push notifications to mark important baby developmental milestones and special parenting tips. A study that investigated the use of a technology-based supportive intervention among multiracial Singaporean mothers during the postpartum period suggested the extension of the parenting support programs beyond 1 month after childbirth, introducing the parenting app early in pregnancy, and including a greater variety of educational topics [39].

### Rigor Cycle

To meet the goals of the rigor cycle, well-established theoretical frameworks were incorporated into the development process of the SPA. The research team referred to the mHealth user engagement pyramid proposed by Singh et al [41] to identify features that could be incorporated into the SPA. Providing educational information is the most fundamental way to engage a patient, and the level of engagement increases with add-on features such as reminders and notifications, records of health information, display and summary of health information, availability of individualized guidance, means of communication between clinicians and family members or caregivers, provision of support through social networks, and incentivization of behavioral changes [41].

Other theoretical frameworks that guided the development of the SPA’s features were the social cognitive theory proposed by Bandura [42,43] and the attachment theory proposed by Bowlby [44]. The social cognitive theory posits that an individual’s knowledge acquisition depends on the interaction among the environment, cognitive behavior, and personal factors [43]. An important component of the social cognitive theory is self-efficacy, which is defined as one’s belief in one’s capability to accomplish a task effectively [42]. Therefore, to ensure successful parenting, Bandura [42,45] emphasized that parents need to possess the self-confidence to perform specific skills and believe that they can achieve their desired outcome. As evidence has shown low levels of parenting self-efficacy as a predictor of postpartum depression [46], the concepts of the self-efficacy theory proposed by Bandura [42,45] were incorporated into the development of the SPA. Mastery of experiences, vicarious experiences, verbal persuasion, and improving physical and emotional states are ways in which self-efficacy can be attained [42]. In addition, Bandura [43] emphasized the importance of social support in achieving self-regulation to aid learning. There are different types of social support (emotional, instrumental, informational, and appraisal) [43]. As the SPA is a mobile app intervention, providing instrumental support was not possible; hence, it focused primarily on emotional and informational support. Appraisal support, which involves the provision of feedback regarding one’s performance or qualities, was also not feasible in the context of the SPA. However, specific feedback requested by parents was provided for their individual needs.

**Figure 2:**
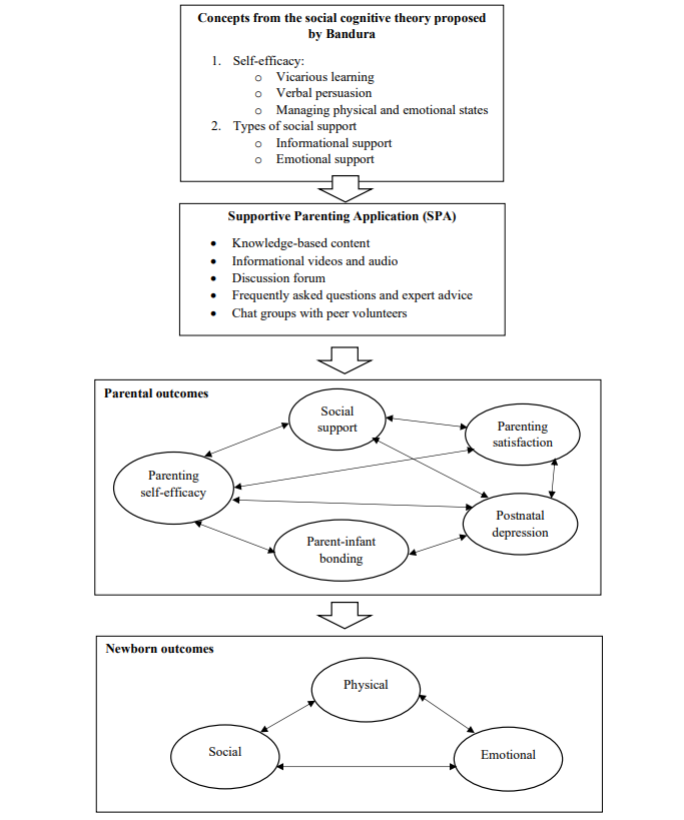
Theoretical framework of the Supportive Parenting App [42,43].

According to the attachment theory proposed by Bowlby [44], parenting self-efficacy, social support, and parental emotional well-being are crucial for early parent-infant bonding and infant attachment styles, and these serve as a foundation for the development of positive social relationships in infants. Bowlby [44] postulated that a healthy parent-infant bond may lead to secure attachment, whereas insecurely attached infants may take their insecure patterns (avoidant, anxious ambivalent, or disorganized) with them into adulthood. Congruent to the attachment theory proposed by Bowlby [44], Bandura [45] postulated that self-efficacy will consequently influence parent-infant bonding, which is an important determinant of parental emotional well-being and infant development. Therefore, it is necessary to examine the relationship among parenting self-efficacy, parental outcomes, and infant outcomes. The theoretical framework for this project is presented in Figure 2 [42,43].

### Design Cycle: Development Phase

#### Overview

The research team involved in the development of the SPA comprised maternity health care providers such as obstetricians, midwives, and nurses from the tertiary public hospitals; a psychologist; a pediatrician; and app developers. The intervention consisted of 2 main components: (1) a cloud-based admin portal, which was fully accessible by system admins (midwives and physicians only had access to the frequently asked questions [FAQ] management page) and (2) the SPA, which was accessible by system admins, mothers, fathers, peer volunteers, midwives, and physicians.

The cloud-based admin portal was used to moderate the content in the FAQ section and chat groups, manage user accounts and surveys, and display simple user analytics. The admin portal was developed using Microsoft Visual Studio 2017. The main programming languages used for the back end were ASP.NET and C#, running on .NET Framework (version 4.5 and above; Microsoft Corporation). The front-end languages used were HTML5, JavaScript, CSS, and jQuery. AJAX techniques were used across the system to facilitate asynchronous data transfer between client and server. The database for the system was Microsoft SQL Server 2016 or later. The system was hosted on Windows Server 2016 or above and Internet Information Services (version 10.0 or above).

The SPA ran on both iOS and Android built using hybrid development tools (Apache Cordova and Ionic Frameworks). All accounts were password protected, and users had to authenticate with their log-in credentials created by the system admin to use the app. Participants were assigned anonymous identifiers, and they remained anonymous throughout.

**Figure 3:**
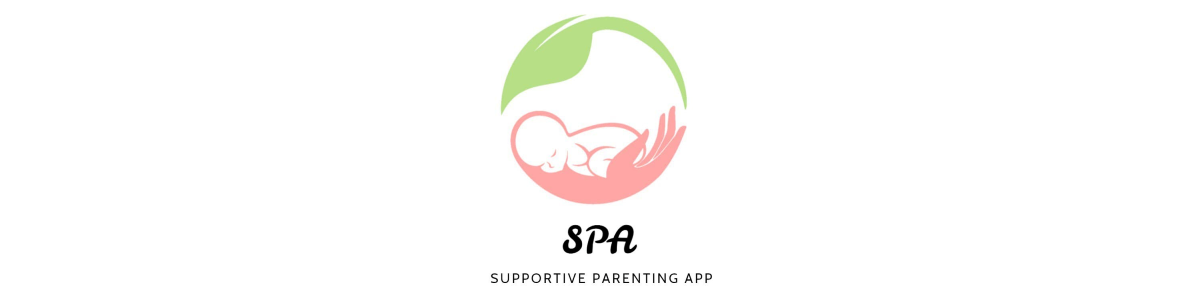
Logo of the Supportive Parenting App.

After the health care research team conveyed the expectations, aims, and thorough overview of the SPA project to the app developers, a logo for the SPA was generated. A pastel-colored theme was chosen to create a soothing and gentle vibe, and the logo displayed an infant lying in the nurturing palm of a parent. The app’s aim to highlight the protective and nurturing nature of parents was also demonstrated by the green sprout emerging from the parent’s hand that sheltered the infant. The design was finalized after multiple intense discussions between the app developers and the research team; the logo is shown in Figure 3.

The main features of the SPA included knowledge-based content, informational videos and audio clips, a discussion forum, group and private chats with peer volunteers, expert advice from a maternity unit nurse or midwife, and individualized push notifications. Details of the development of each component are described in the following sections.

#### Knowledge-Based Content

According to Bandura [42,43], the provision of informational support is useful for problem solving and knowledge acquisition. To provide comprehensive information to allay parents’ concerns in the perinatal period, knowledge-based content was first brainstormed and then finalized after multiple rounds of intense discussions among a team of health care experts from the psychology, pediatrics, and maternity health care units, as well as a few parent couples. The discussions were held through face-to-face and web-based meetings.

**Figure 4:**
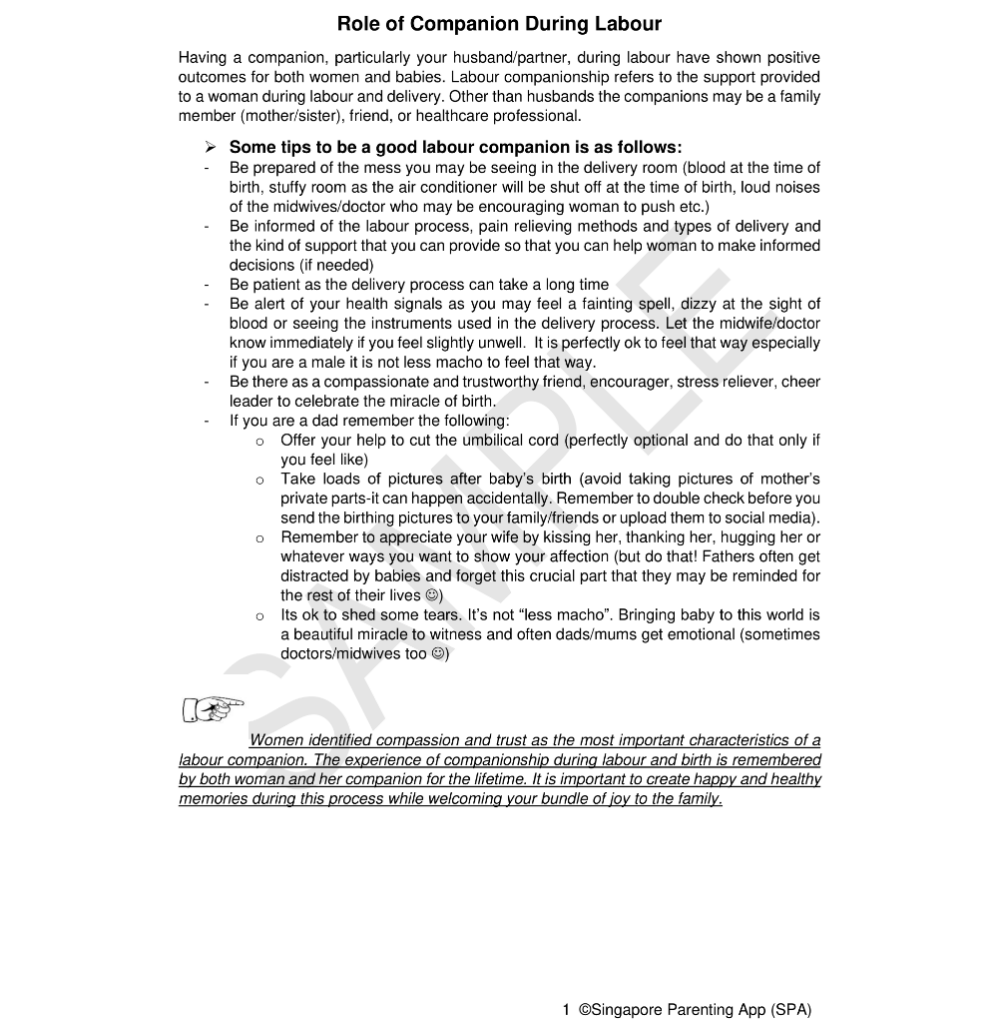
Sample of a knowledge-based article.

The finalized topics were categorized into 6 groups (general, pregnancy, childbirth, baby care, maternal care, family, and parenthood) to enable users to obtain relevant information. Keyword search was enabled, and the content was sorted by most viewed or most rated. Parents were also allowed to rate the usefulness of the content on a scale of 1 to 5. Knowledge-based content included topics on the prevalence of postpartum depression among mothers and fathers, signs and symptoms of postpartum depression, when to seek help, diet and exercise during pregnancy and after childbirth, preparation for childbirth, types of pain relief during childbirth, newborn and postpartum maternal care, major milestones for the newborn, managing family dynamics, and role of fathers and other caregivers (eg, grandparents) during the perinatal period. There were a total of 42 knowledge-based articles; each knowledge-based article was approximately 500 words long, and the content was written in bullet points to facilitate reading. The *hand symbol* was used consistently to signpost additional parenting tips or highlight instances when parents need to seek emergency help. The English language used was simplified to that of the reading ability of students in primary 6, and this was verified by the publications support unit at the National University of Singapore. An example of a knowledge-based article is shown in Figure 4.

#### Informational Videos and Audio Clips

**Figure 5:**
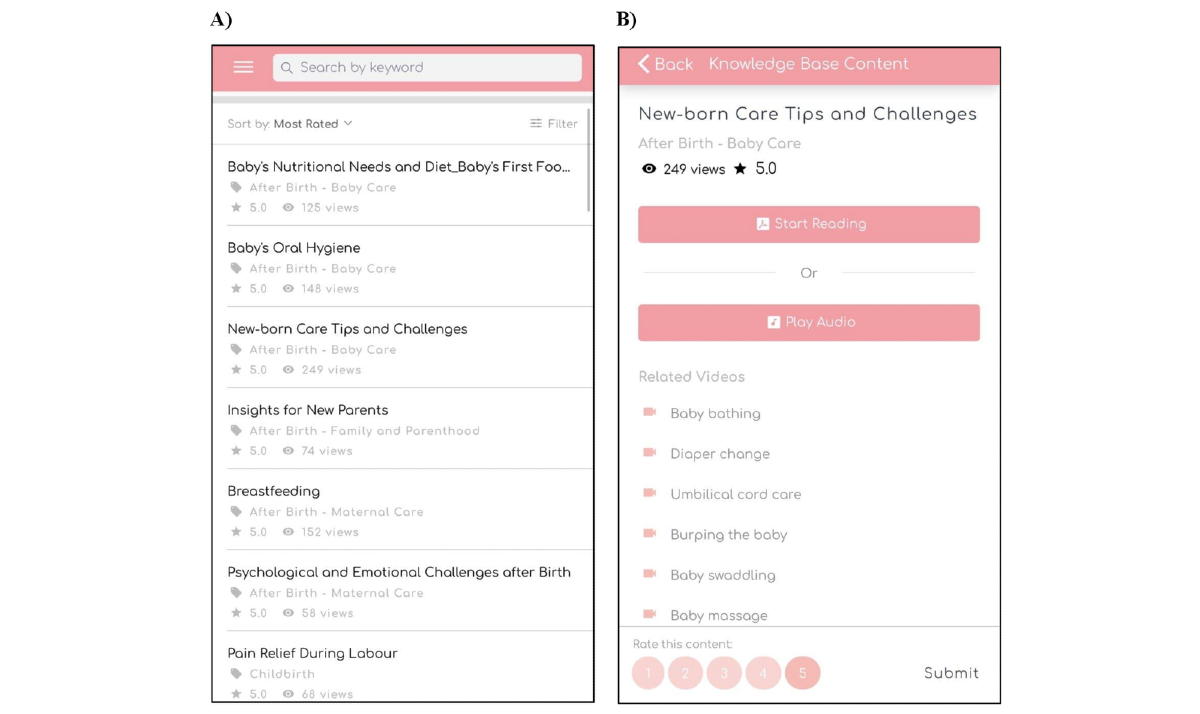
(A) Knowledge-based content page with (B) video and audio materials.

Parenting self-efficacy can be enhanced through vicarious learning or modeling by watching others or personally acquiring a certain behavior successfully [42]. Hence, 27 inspirational and demonstration videos were included in the knowledge-based content section. Video content and scripts were developed by health care experts from different fields. For instance, baby care video scripts were developed by health care professionals from the maternity unit and video scripts on insights into postpartum depression were prepared by psychology experts. The videos consisted of animations, interviews, and skills demonstrations of topics such as personal accounts of mothers who developed and recovered from postpartum depression, diet and exercise during pregnancy, preparation for childbirth, and demonstration of infant care skills and interviews related to a father’s role during the perinatal period. Audio versions were also provided to cater to parents who preferred to listen to audio clips or were prohibited from reading or watching videos because of confinement practices. A screenshot of the knowledge-based content page, including videos and audio clips, is shown in Figure 5.

#### Discussion Forum

**Figure 6:**
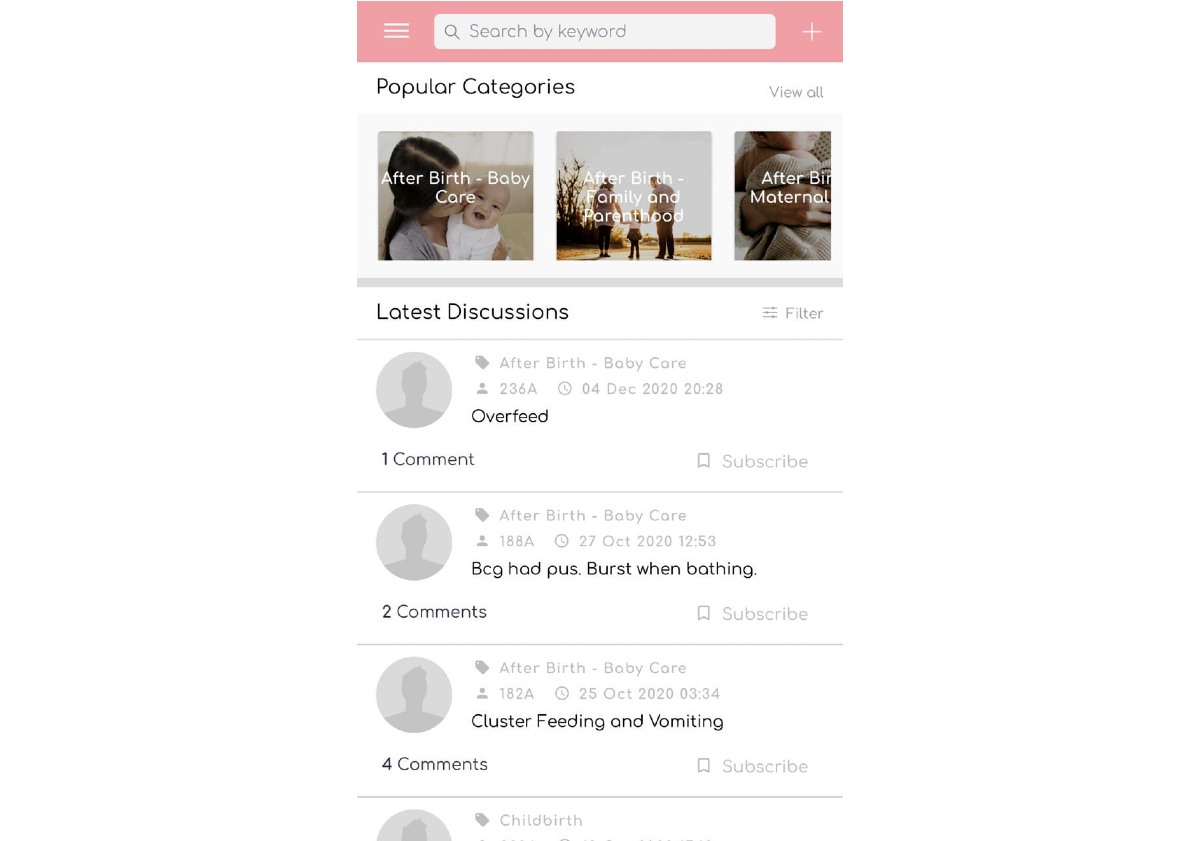
Discussion forum in the Supportive Parenting App.

Apart from video modeling, a discussion forum was developed to allow parents who faced similar problems to share their tips and experiences. Parents were able to post personal queries and respond to other parents’ queries. A midwife or maternity unit nurse was able to moderate the comments to ensure the reliability and accuracy of the information shared. A sample of the discussion forum is shown in Figure 6.

#### FAQ and Expert Advice

**Figure 7:**
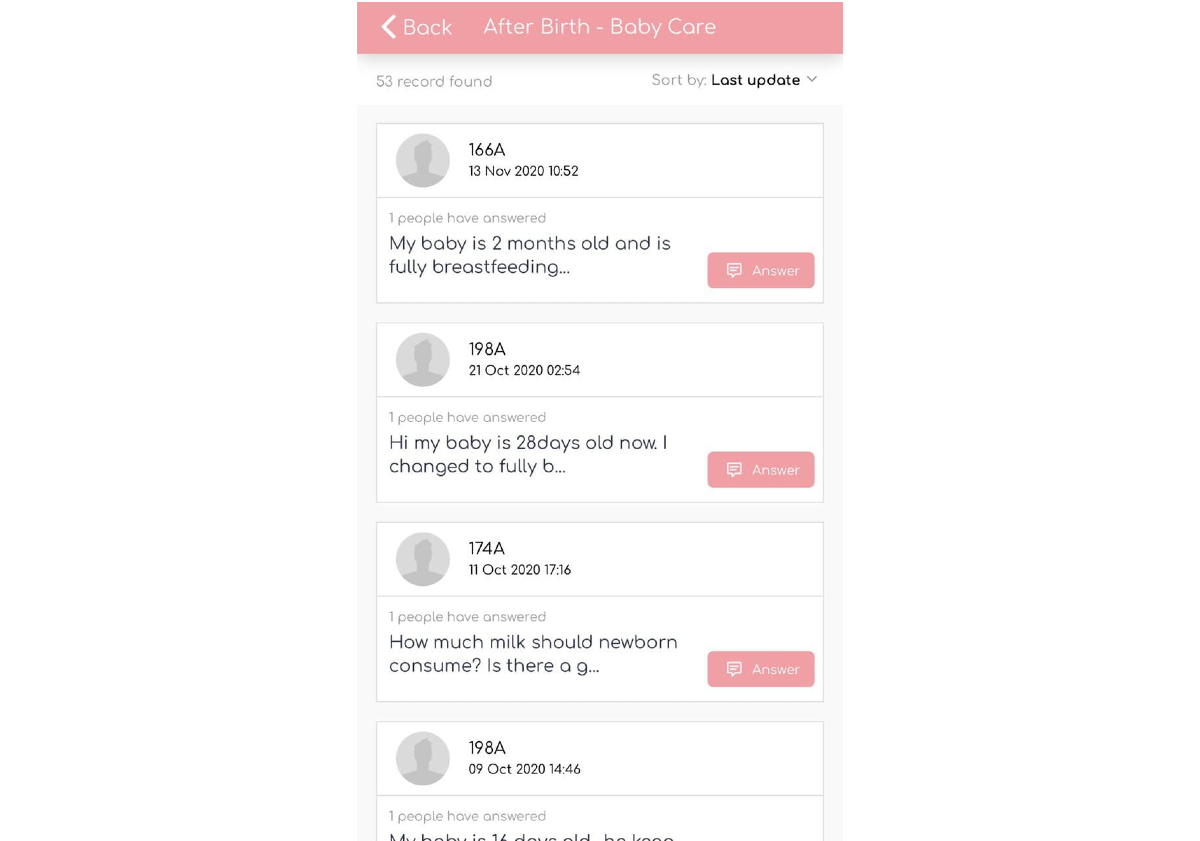
Frequently asked questions and experts’ advice page.

Further informational support by health care professionals was provided through an FAQ section. If the existing information was insufficient to answer the parents’ queries, parents were able to post their questions and upload supporting photos. A screenshot of the FAQ and expert advice section is shown in Figure 7. This feature allowed individualized professional help without parents having to make unnecessary visits to the physician. Expert advice from the maternity unit nurse or midwife was available from pregnancy until 1 month after childbirth. Because of the busy work schedules of the maternity unit nurse and midwife, replies were posted once a day.

#### Chat Groups With Peer Volunteers

Mothers were matched for variables not limited to ethnicity and number of children and then assigned to peer volunteers. They were able to seek informational and emotional support from peer volunteers and other mothers or have a private chat with their assigned peer volunteer in the chat groups. Peer volunteers were experienced mothers who had developed and recovered from postpartum depression previously. These peer volunteers were specially trained by a psychiatrist on how to provide adequate support and be a listening ear to needy mothers, as well as on how to navigate the SPA. To ensure fidelity, an electronic training manual was provided to all peer volunteers before the start of a 3-hour web-based training session, and skills acquisition was assessed by the psychologist through web-based role-playing. After the training, each peer volunteer was assigned to approximately five couples (fathers and mothers) who were added to a group chat. Peer volunteers were encouraged to check up on the participants regularly through the group chat and to encourage mothers to interact and share their experiences. Parents who were uncomfortable with expressing themselves in a group setting had the choice to send private messages to their peer volunteer. Overall, the role of the peer volunteer was to provide emotional support to needy mothers, help foster peer support, and develop a close-knit community among parents in the chat group.

#### Push Notifications

**Figure 8:**
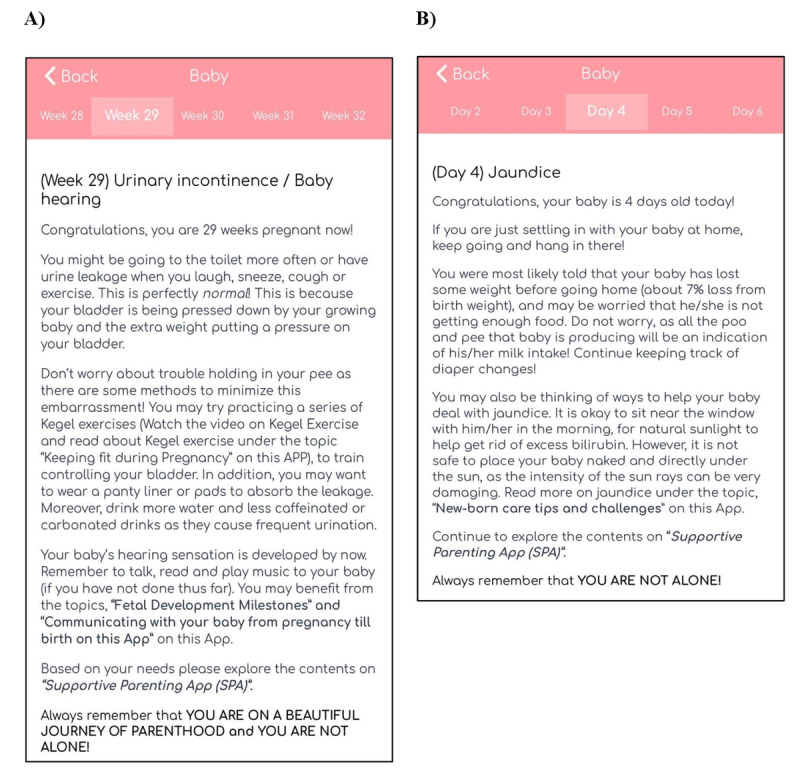
Examples of push notifications delivered (A) during pregnancy and (B) after childbirth.

Finally, push notifications were sent on a weekly basis during pregnancy, daily during the first month of the postpartum period, and biweekly thereafter until 6 months after childbirth. These notifications provided individualized information to parents, according to the specific week of their pregnancy and the age of their newborn after childbirth. The push notifications during pregnancy helped to track the pregnancy stages and provided information specific to the mother’s pregnancy stage, such as common physical experiences (eg, Braxton Hicks contraction), ways to manage physical symptoms, relevant informational material that parents could refer to in the SPA, and words of encouragement. Push notifications that were sent after childbirth kept both parents updated on the infant’s developmental milestones, informed parents on common physical occurrences and changes in the mother (eg, vaginal bleeding) and the infant (eg, weight loss), presented ways to care for both mother and infant, provided supportive tips for fathers, guided parents to relevant information in the SPA, and included words of encouragement. Parents were also advised to look out for required information from the knowledge-based content and informational videos and audio clips according to their individualized needs and not to switch off the notifications because this could interfere with the intervention. Examples of push notifications delivered during pregnancy and after childbirth are presented in Figure 8.

### Design Cycle: Evaluation

#### Pilot Test

To assess the SPA’s features, functionality, usability, and content accuracy, a pilot test for the mobile app and administrator webpage was conducted among users (N=10) who were new parents and research team members (ie, app developers, clinicians, and research assistants). Feedback was gathered qualitatively and discussed among team members at a web-based meeting to further refine the app and optimize the user experience. This process was repeated multiple times and ceased when all the research team members arrived at a mutual agreement and were satisfied with the finalized version of the app. The mobile app was then made available to participants in the iOS and Android app stores.

#### System Compatibility

During the initial piloting, there were issues regarding the viewer compatibility of the cloud-based admin portal on different web browsers. The admin portal was not accessible on Internet Explorer and could only be viewed using Google Chrome, Mozilla Firefox, or Microsoft Edge. This was subsequently made known to all system admin users. Similarly, the SPA was initially incompatible with the older Android OS versions, but updates were made by the developer to support the older OS versions.

#### Restricted Admin Functions and Data Analytics

In addition, the admin portal did not allow for a full overview of and access to the SPA’s contents, especially in terms of group chat details. System admin users could only view peer volunteer group chat conversations using the mobile app. In addition, the initial data analytics function on the admin portal only displayed aggregated data on user frequency. However, individual user frequency data were deemed more informative for future research purposes. As subsequent requests for collating individual user data by the research team resulted in extra development charges and time, no enhancement was made to the data analytics.

#### App Notifications

The first testing of the SPA revealed that mothers did not receive group chat notifications. This was quickly fixed by the app developers so that mothers could receive a pop-up notification whenever somebody posted in the group chat. However, the problem persisted in the second round of user testing. Group chat notifications were not received when the app was not constantly updated, and parents did not receive any prompt to update the app. This situation was later rectified by sending reminders to update the app. Similarly, team members in charge of the expert advice section made a request for pop-up notifications to enable them to respond to parents’ queries promptly. However, this was not implemented because of the project’s tight budget. After the first user testing, educational contents (PDF files and videos) were not linked to the push notifications as expected. This issue was then rectified by the app developers for parents’ convenience.

#### Search Function

The research team also highlighted issues with the search function of the knowledge-based content. Initially, the search engine was only able to detect specific keywords in the title but not in the text. The function for in-text search required the additional purchase of plug-ins and further development of codes that involved time and cost that the team did not take into account initially.

## Discussion

### Principal Findings

The development process of the SPA, which included requirement identification, the use of theories and past artifacts, the design of artifacts, and user testing, was well documented using the ISR framework. Although the ISR framework was able to capture the design technicalities and rigor of the project, it failed to highlight interpersonal and real-life challenges. Apart from technical and user issues, the design cycle faced prominent issues such as logistical limitations and also in terms of teamwork and communication because of the effects of the COVID-19 outbreak, which affected the process timeline.

### Challenges in the Design Cycle: Logistics Limitations

With respect to the FAQ and expert advice section, the research team ideally wanted to provide a readily available hotline or personalized help for parents in need. However, because of limited resources and other work commitments, it was not feasible for the research team to be on constant standby to respond to parents’ queries immediately. Hence, a decision was made to have a maternity unit nurse or midwife to check and respond to queries posted in the FAQ section once a day and respond within 24 hours of the queries raised by the parents. Parents with urgent queries were recommended to call the hospital or visit the physician immediately. Despite the team’s best efforts to provide parents with a personalized, fast, trusted, and reliable information source, the strain on logistics and manpower led the research team to consider the future incorporation of live chatbots in the mobile app. The use of chatbots or conversational agents in the health care setting has been on the rise, especially with smartphone apps, in the areas of patient treatment and monitoring, health care service support, and patient education [47]. Given the novelty of the use of chatbots, there is a paucity of randomized controlled trials evaluating their efficacy in providing knowledge-based information to parents in the perinatal period. However, promising user test results from a few development and usability studies [48-50] indicate the viability of chatbots, thus paving the way for future research.

Second, the recruitment and training of peer volunteers posed a challenge because peer volunteers were also mothers who had work and family commitments; hence, it was difficult to recruit dedicated peer volunteers to take on this yearlong project. In addition, the conflicting schedules of peer volunteers resulted in a few postponements of face-to-face training sessions until the COVID-19 outbreak led to their conversion to web-based sessions instead. Team members in charge of conducting the sessions had to rapidly adapt to the situation and convert the face-to-face training to a web-based session. Research has shown that the efficacy of web-based interpersonal skills (ie, communication, counseling, and interview) training on skills acquisition is on par with face-to-face training [51-53]. Furthermore, participants have also commended web-based courses for their better accessibility and the ease of remote learning [54,55]; thus, a web-based session may better cater to volunteers who are working mothers. However, Doo [56] has argued that cognitive understanding of interpersonal skills does not translate into successful skills execution and interpersonal skills can only be acquired through real-life practice, which is difficult in web-based learning environments. Although web-based training may not be as beneficial as in-person training in terms of building rapport among peer volunteers and conducting role-plays, this was the optimal resolution in times of a COVID-19 outbreak. We are waiting to interview the peer volunteers at the completion of this project about their perceptions of receiving the web-based training. The interviews may provide true evaluation and recommendations on enhancing such web-based training in the future.

### Challenges in the Design Cycle: Teamwork and Communication

As this was intended to be a multisite study, extensive collaboration among health care professionals from various institutions was required. However, a key challenge faced during the Tuckman model’s forming phase of team development [38] was the assembling and scheduling of face-to-face meetings with different experts from various health care institutions, given their hectic schedules. As face-to-face meetings were crucial at the initial stage for the research team to get acquainted and share their expertise and viewpoints, meeting dates were scheduled when there was a likelihood of the highest possible attendance. Team members who were unable to attend received the meeting materials and minutes, and any further feedback was communicated through email. After the delegation of the main roles and responsibilities, subsequent follow-up meetings, updates, and discussions were conducted through web-based meetings with respective parties. At the storming phase, given each team member’s field of expertise and the dearth of essential information, it was difficult for the team to agree on the finalized topics and knowledge-based content to be included in the app. However, the team managed to ascend to the norming phase through resolution by constructive negotiation and eventual mutual agreement to include less essential information in the FAQ section than in the knowledge-based content section. During the performing phase, the team managed to work together effectively and cohesively and successfully rolled out the SPA for user acceptability testing. Overall, the Tuckman model of team development highlighted key characteristics and conflict resolution strategies that enabled optimal team development, which future mobile app developmental studies can use.

Another prominent issue concerned the communication between the app developers and the health care research team. Although the research team comprised experts in content development, they were unfamiliar with technical programming terms and were unable to communicate effectively with the app developers on their app preferences such as cloud hosting, data analytics function, and security mechanisms of the app. This communication difficulty resulted in different expectations for the app and led to last-minute enhancements that incurred additional costs that were not initially considered. Therefore, both parties should be careful with the use of technical jargon to avoid confusion and to constantly clarify any uncertainty or details. Alternatively, visual diagrams (eg, mind maps, drawings, and sample screenshots) could be useful in visualizing and conveying expectations of specific mobile app features.

### Implications for Future Studies

Much can be learned from the intervention development process of the SPA based on the aforementioned challenges. A major setback was the sudden COVID-19 outbreak, which interrupted the development of the intervention and delayed the project. The flexibility and quick adaptability of the team in finding solutions was crucial in ensuring the continuity of the project. Although face-to-face meetings were impossible, the team was able to conduct meetings through web-based video calls and follow-up emails. Face-to-face training for peer volunteers was also revised and conducted on the web. Team cohesiveness and adaptability were crucial in effectively managing unforeseen circumstances.

Team cohesion is dependent on open and clear communication among team members to ensure that everyone’s expectations are aligned, uncertainties are clarified, and there is transparency for group decision-making. This is especially crucial between the research team and the app developers because their areas of expertise differ and miscommunication in expectations could lead to unfavorable outcomes. However, the interdisciplinary, team-based approach ensured the success of the app development in this study. In addition, a structured and effective workflow was implemented through strict planning of timelines, delegation of responsibilities to team members, close updates and timely feedback among team members, and the charting of project milestones using the Gantt chart. The physical visualization of the project timeline kept everyone in check and allowed better handling of delays.

Another critical lesson learned was to ensure sufficient financial and time budgeting for last-minute enhancements of the intervention. Because of project delays during the COVID-19 pandemic, the team could not spend more time and money for potential feature enhancements that may influence the detailed data analytics. As the basic infrastructure for the parenting app has already been established and the team members have acquired sufficient experience, incorporating future improvements such as adding additional features to the platform would require much less time than that needed for the initial project. Future studies developed as part of this SPA project could assess actual app use, cost-effectiveness, impacts on longitudinal parental and infant outcomes, and qualitative user experience. The use of chatbots to answer parental queries on a timely basis could also be incorporated and evaluated in future research on supportive apps for parents.

### Conclusions

This study presents a multistage iterative development process of an mHealth SPA using the ISR framework. Rooted in social cognitive theory and attachment theory, the SPA offers parents a holistic means of obtaining informational and emotional support from knowledge-based content, parents with similar backgrounds, trained peer volunteers, and health care professionals. However, the development process had its challenges because the design cycle involved other logistical and team communication issues that were not taken into consideration in the ISR framework. These issues can potentially be resolved with adequate time and financial budgeting and by developing team cohesion.

## Abbreviations

FAQ: frequently asked questions
ISR: information systems research
mHealth: mobile health
SPA: Supportive Parenting App
